# A coupled algebraic-delay differential system modeling tick-host interactive behavioural dynamics and multi-stability

**DOI:** 10.1007/s00285-023-01879-8

**Published:** 2023-02-04

**Authors:** Xue Zhang, Jianhong Wu

**Affiliations:** 1 Department of Mathematics, Northeastern University, Shenyang, 110819 People’s Republic of China; 2 Laboratory for Industrial and Applied Mathematics, York University, Toronto, ON M3J 1P3 Canada

**Keywords:** Algebraic-delay differential equation, Structured tick population dynamics model, Host grooming behaviour, Multi-stability, 92D95, 34A09, 34D20

## Abstract

We propose a coupled system of delay-algebraic equations to describe tick attaching and host grooming behaviors in the tick-host interface, and use the model to understand how this tick-host interaction impacts the tick population dynamics. We consider two critical state variables, the loads of feeding ticks on host and the engorged ticks on the ground for ticks in a particular development stage (nymphal stage) and show that the model as a coupled system of delay differential equation and an algebraic (integral) equation may have rich structures of equilibrium states, leading to multi-stability. We perform asymptotic analyses and use the implicit function theorem to characterize the stability of these equilibrium states, and show that bi-stability and quadri-stability occur naturally in several combinations of tick attaching and host grooming behaviours. In particular, we show that in the case when host grooming is triggered by the tick biting, the system will have three stable equilibrium states including the extinction state, and two unstable equilibrium states. In addition, the two nontrivial stable equilibrium states correspond to a low attachment rate and a large number of feeding ticks, and a high attachment rate and a small number of feeding ticks, respectively.

## Introduction

Tick-borne diseases (TBDs) have been imposing significant public health challenges globally. The TBD transmission relies on the tick-host interaction where ticks may acquire and/or transmit the pathogen from the host by taking a blood meal from the host. There are systemic transmission and co-feeding routes, both depending on the co-occurrence of ticks (specially ticks at two different stages for co-feeding transmission to take place) on the same host, so understanding the tick load distribution dynamics over the host and its implication for the tick population dynamics, the main focus of this study, is important. The tick load distribution process over the host is a dynamical process, governed by the tick attachment/fixation and host grooming behaviours. Our study shows that these tick and host individual behaviors, the host response to tick attaching and/or to tick feeding, and their combinations can yield a complicated tick-host interaction leading to multi-stability where tick densities can converge to a tick extinction, or a lower level tick persistence, or a higher level tick persistence equilibrium state depending on the initial conditions.

Tick life cycle includes four stages: egg, larvae, nymph and adult. Larval and nymphal ticks seek blood meals from small rodents, like mice and bird, to molt into nymphal and adult stage, respectively. While it is hard to find larval and nymphal ticks on the hosts since they are small [only around 1–2 mm in size (Lindquist and Vapalahti 2008)], ticks at these stages are very important for the tick-borne pathogen transmission as vertical transmission (from egg-laying infected ticks to eggs) are limited. Adult ticks prefer questing large mammals, such as deer and domestic livestock, both infected nymphal and adult ticks can bite on human, passing the pathogen to the human. After the final blood meal, adult tick will lay egg to complete the life cycle from eggs to egg-laying ticks.

Important to the survival of an egg through the life cycle is the attachment/fixation success of the questing tick. Existing models have assumed a constant attachment success rate, so the fixation rate of an engorged tick to a host for feeding to develop into the next stage is the questing rate times the attachment/fixation success rate. However, it was reported (Voordouw 2015; Wang et al. 1998, 2001; Rechav and Nuttall 2000) that ticks may pool their saliva to enhance the immunomodulatory manipulation of the host organism. The resulted *cooperative feeding* could increase the cost-benefit ratio of resource extraction from the host relative to per capita investment in tick saliva production. Reflecting this cooperative feeding requires a (feeding tick) density-dependent attachment/fixation rate, as we will consider in our model formulation.

Equally important to the survival of an egg through the life cycle is the grooming behavior of the host, leading to (feeding tick) density-dependent grooming rate (or feeding tick survival rate) in some of the proposed mathematical models. Pioneering observation of the phenomenon called *acquired tick resistance (ATR)*, by Trager (1939), showed that upon repeated tick infestations, hosts develop an immune response to derail subsequent tick challenges, and tick-immune hosts rapidly reject ticks within the first 24 h of tick attachment. See recent reviews (Narasimhan et al. 2021; Yoshikawa et al. 2020) for the current knowledge of ATR and key events in the tick-host interaction to enable or disable tick feeding. Evidence provided by Hart and his colleagues (Hart 2000) provided support to “the concept that the delivery of bouts of grooming reflects programmed grooming”, namely, “grooming occurs in response to an endogenous generator that produces grooming bouts at periodic intervals, resulting in removing of ticks before they attach and begin to feed”. Correspondingly, in our model formulation, we will consider (feeding tick) density-dependent grooming and/or attachment/fixation-dependent grooming rate.

In summary, a mathematical description of the tick population dynamics must take consideration of the tick attachment and fixation dynamics, and host grooming dynamics. Here, we develop a coupled system involving two state variables, the number of engorged nymphal ticks and the number of feeding nymphal ticks. We start with an evolution equation for the feeding tick density (with respect to the time since feeding nymphal ticks attach to the host) governed by the density-dependent grooming rate, and subject to an initial condition involving density-dependent attachment and fixation rate. Integration along a characteristic equation leads to an algebraic equation for the total feeding (nymphal) ticks. We then couple this algebraic equation with a delay differential equation for the engorged nymphal tick dynamics. Since questing nymphal ticks come from the engorged nymphal ticks with a delay after further development and production, the coupled system becomes a closed feedback system with delay.

The model will be formulated in Sect. 2; the model’s equilibrium structure is described in Sect. 3; and the stability of equilibrium states is discussed in Sect. 4 using a perturbation argument since the feeding duration is relatively short in comparison with the life cycle. Additional discussions on how this tick population dynamics can be extended to model tick-borne disease transmission dynamics involving the co-feeding transmission route are provided in the final section.

## The algebraic-delay coupled system

We focus on the developmental stage, nymphal stage, where questing and attaching rate of the ticks and the grooming rate of the hosts may depend on the feeding nymphal tick loads on the host.

### The model derivation and simplification

Let *Q*(*t*), *F*(*t*) and *E*(*t*) be the numbers of questing nymphal ticks, feeding nymphal ticks and engorged nymphal ticks, respectively, at time *t*. We make it explicit our standing assumptions:

(i) The attachment rate of questing nymphal ticks is a function $\rho (F(t))$ of the number of feeding nymphal ticks *F*(*t*). This is to reflect the fact that the attachment success of questing ticks depends on the total amount of feeding nymphal ticks on the hosts. As the total number of hosts for nymphal ticks is relatively static, the average feeding ticks on the hosts is proportional to *F*(*t*). In what follows, we assume $\rho :[0, \infty )\rightarrow [0, \infty )$ is a $C^1$-smooth function. Moreover, $\rho (F)>0$ if $F>0$.
(ii) The drop off rate of feeding nymphal ticks is a function $\nu (F(t))$ of the number of feeding nymphal ticks *F*(*t*). This is to describe the host grooming behaviors, and can also be used to describe the cooperative co-feeding behaviours of the nymphal ticks (that enhances the immune-induced feeding). We also assume that $\nu :[0, \infty )\rightarrow [0, \infty )$ is a $C^1$-smooth function, and $\nu (F)>0$ if $F>0$.

Let *n*(*t*, *a*) be the density of feeding nymphal ticks at time *t* with respect to feeding duration *a* since they attach to the host. Then we have the structured feeding tick population dynamics model

1 $$\begin{aligned} \begin{aligned}&\left( \dfrac{\partial }{\partial t}+\dfrac{\partial }{\partial a}\right) n(t,a)=-\nu (F(t))n(t,a),~~ 0\le a\le T,\\&n(t,0)=\rho (F(t))Q(t), \end{aligned} \end{aligned}$$

where *T* is the average feeding duration of nymphal ticks. In this formulation, the evolution describes the grooming dynamics while the boundary condition describes the attaching behaviours.

The structured population model can be easily solved using the method of integration along characteristics (Evans 1998). Namely, let

2 $$\begin{aligned} \zeta (t)=n(t,t-s), ~~ t\ge s, \end{aligned}$$

then we can rewrite (1) into the following form

3 $$\begin{aligned} \begin{aligned} \dfrac{d\zeta (t)}{dt}&=-\nu (F(t))\zeta (t),~ t\ge s,\\ \zeta (s)&=n(s,0)=\rho (F(s))Q(s). \end{aligned} \end{aligned}$$

Integration of (3) yields

$$\begin{aligned} \zeta (t)=e^{-\int _s^t\nu (F(\theta ))d\theta }\zeta (s)=e^{-\int _s^t\nu (F(\theta ))d\theta }\rho (F(s))Q(s), \end{aligned}$$

which, from the definition of (2), is equivalent to

$$\begin{aligned} n(t,t-s)=e^{-\int _s^t\nu (F(\theta ))d\theta }\rho (F(s))Q(s). \end{aligned}$$

Setting $t-s=a$, we obtain

$$\begin{aligned} n(t,a)=e^{-\int _{t-a}^t\nu (F(\theta ))d\theta }\rho (F(t-a))Q(t-a). \end{aligned}$$

Clearly, the total amount of feeding nymphal ticks can be expressed as the integral of *n*(*t*, *a*) over the feeding interval, i.e.,

$$\begin{aligned} \begin{array}{ll} &{}F(t)=\int _0^Tn(t,a)da=\int _0^Te^{-\int _{t-a}^t\nu (F(\theta ))d\theta }\rho (F(t-a))Q(t-a)da\\ &{}\quad \;\;\;\; =\int _0^Te^{-\int _{-a}^0\nu (F(t+\xi ))d\xi }\rho (F(t-a))Q(t-a)da, \end{array} \end{aligned}$$

which is an implicit equation for *F*(*t*). Then the dynamics for engorged nymphal ticks follows

$$\begin{aligned} \begin{aligned} \dfrac{dE(t)}{dt}&=-\delta E(t)+n(t,T)\\&=-\delta E(t)+e^{-\int _{t-T}^t\nu (F(\theta ))d\theta }\rho (F(t-T))Q(t-T), \end{aligned} \end{aligned}$$

where $\delta$ is the exit rate of engorged nymphal ticks.

We now link the questing nymphal ticks at the current time to engorged nymphal ticks in the past through the life cycle. Let $\eta _1$ be the survival probability from engorged nymphal ticks to adult egg-production ticks, $\sigma$ be the egg production rate, and $\eta _2$ be the survival probability from eggs to questing nymphal ticks. Assume that $\tau _2$ represents the delay from eggs to questing nymphal ticks through the necessary developments and $\tau _1$ is the delay from engorged nymphal ticks to adult egg-laying ticks. Hence, questing nymphal ticks at time *t* take the following form

$$\begin{aligned} Q(t)=\eta _2\sigma \eta _1 E(t-\tau _1-\tau _2). \end{aligned}$$

For simplicity, we denote $\tau =\tau _1+\tau _2$ and $\eta =\eta _2\sigma \eta _1$. From the definition of feeding duration *T* of nymphal ticks, it is easy to see that the life cycle of tick population is $T+\tau$. We obtain the following coupled system to describe the dynamics of feeding and engorged nymphal ticks

4 $$\begin{aligned} \left\{ \begin{aligned} \dfrac{dE(t)}{dt}&=-\delta E(t)+e^{-\int _{t-T}^t\nu (F(\theta ))d\theta } \rho (F(t-T))\eta E(t-\tau -T),\\ F(t)&=\int _0^Te^{-\int _{-a}^0\nu (F(t+\xi ))d\xi } \rho (F(t-a))\eta E(t-\tau -a)da. \end{aligned} \right. \end{aligned}$$

### Fundamental theory

We now show that the above coupled system of differential-algebraic system is equivalent to a coupled system of delay differential equations subject to a matching condition of the initial data.

Differentiating the right-hand side of the algebraic equation for *F*(*t*), we get

5 $$\begin{aligned} \dfrac{dF(t)}{dt}=\,&\dfrac{d}{dt}\int _0^Te^{-\int _{-a}^0\nu (F(t+\xi ))d\xi } \rho (F(t-a))\eta E(t-\tau -a)da\nonumber \\ =\,&\dfrac{d}{dt}\int _{t-T}^te^{-\int _{\theta }^t\nu (F(s))ds} \rho (F(\theta ))\eta E(\theta -\tau )d\theta \nonumber \\ =\,&\rho (F(t))\eta E(t-\tau )-e^{-\int _{t-T}^t\nu (F(s))ds}\rho (F(t-T))\eta E(t-T-\tau )\nonumber \\&-\int _{t-T}^te^{-\int _\theta ^t\nu (F(s))ds}\nu (F(t))\rho (F(\theta ))\eta E(\theta -\tau )d\theta . \end{aligned}$$

This is equivalent to the algebraic equation if the following matching condition is met:

6 $$\begin{aligned} F(0)=\int _0^Te^{-\int _{-a}^0\nu (F(\xi ))d\xi } \rho (F(-a))\eta E(-\tau -a)da. \end{aligned}$$

Now, we can use the fundamental theory for functional differential equations (Hale 1977) to conclude that for any $(\phi , \psi )\in C([-\tau -T,0];[0,+\infty ))\times C([-\tau -T,0];[0,+\infty ))$, there is one and only one solution of the following coupled system of delay differential equations

$$\begin{aligned} \left\{ \begin{aligned} \dfrac{dE(t)}{dt}=&-\delta E(t)+e^{-\int _{t-T}^t\nu (F(\theta ))d\theta } \rho (F(t-T))\eta E(t-\tau -T),\\ \dfrac{dF(t)}{dt}=&\rho (F(t))\eta E(t-\tau )-e^{-\int _{t-T}^t\nu (F(s))ds}\rho (F(t-T))\eta E(t-T-\tau )\\&-\int _{t-T}^te^{-\int _\theta ^t\nu (F(s))ds}\nu (F(t))\rho (F(\theta ))\eta E(\theta -\tau )d\theta . \end{aligned} \right. \end{aligned}$$

The solution $(E(t),F(t))\in R^2$ is defined for $t\ge 0$. With the matching condition (6), we conclude from (5) that

$$\begin{aligned} \frac{d}{dt}[F(t)-\int _0^Te^{-\int _{-a}^0\nu (F(t+\xi ))d\xi } \rho (F(t-a))\eta E(t-\tau -a)da]=0 \end{aligned}$$

and hence *F* satisfies the algebraic equation in system (4).

We now show that if $\phi (\theta ), \psi (\theta )> 0$ for $\theta \in [-\tau -T,0]$, and if the matching condition (6) hold, then $E(t),F(t)\ge 0$ for all $t\ge 0$. To prove this, we use the continuous dependence of solutions on parameter $\epsilon >0$ for the following system:

7 $$\begin{aligned} \left\{ \begin{aligned} \dfrac{dE(t)}{dt}=&-\delta E(t)+e^{-\int _{t-T}^t\nu (F(\theta ))d\theta } \rho (F(t-T))\eta E(t-\tau -T)+\epsilon ,\\ \dfrac{dF(t)}{dt}=&\rho (F(t))\eta E(t-\tau )-e^{-\int _{t-T}^t\nu (F(s))ds}\rho (F(t-T))\eta E(t-T-\tau )\\&-\int _{t-T}^te^{-\int _\theta ^t\nu (F(s))ds}\nu (F(t))\rho (F(\theta ))\eta E(\theta -\tau )d\theta . \end{aligned} \right. \end{aligned}$$

Denote the solution of (7) by $(E^\epsilon (t),F^\epsilon (t))$, $t\ge 0$. If $E^\epsilon (t)\ge 0$, $F^\epsilon (t)\ge 0$ are not true for all $t\ge 0$, then there must be the first $t^*\ge 0$ such that $E^\epsilon (t^*)=0$ and $E^\epsilon (t)>0$, $F^\epsilon (t)>0$ for all $t\in [0,t^*)$. Therefore, we have $\frac{d}{dt}E^\epsilon (t)|_{t=t^*}\le 0$. But using the first equation of (7), we yield

$$\begin{aligned} \dfrac{d}{dt}E^\epsilon (t)|_{t=t^*}\ge \epsilon . \end{aligned}$$

That is a contradiction. Thus, we have the following existence-uniqueness, and positiveness result.

#### Theorem 1

If $(\phi , \psi )\in C([-\tau -T,0];[0,+\infty ))\times C([-\tau -T,0];[0,+\infty ))$, and if the matching condition (6) is satisfied, then system (4) has one and only one solution defined for all $t\ge 0$. This solution is non-negative, namely, $E(t)\ge 0$ and $F(t)\ge 0$ for all $t\ge 0$ if $\phi (\theta ), \psi (\theta )>0$ for $\theta \in [-\tau -T, 0]$.

#### Remark 1

There are two other approaches to establish the fundamental theory for the well-posedness of the coupled system we formulated. First of all, we can solve the algebraic equation by using the implicit function theory to obtain $F(t)=h(E_{[t-\tau -T,t]})$ and then substitute this to the first equation to obtain a single functional differential equation for *E*(*t*) although the right hand side is given implicitly. Alternatively, we can rewrite the algebraic equation as

$$\begin{aligned} \frac{d}{dt}[F(t)-\int _0^Te^{-\int _{-a}^0\nu (F(t+\xi ))d\xi } \rho (F(t-a))\eta E(t-\tau -a)da]=0. \end{aligned}$$

Therefore, the coupled system can be regarded as a special case of the neutral functional differential equation

$$\begin{aligned} \left\{ \begin{aligned}&\dfrac{dE(t)}{dt}=-\delta E(t)+e^{-\int _{t-T}^t\nu (F(\theta ))d\theta } \rho (F(t-T))\eta E(t-\tau -T),\\&\frac{d}{dt}[F(t)-\int _0^Te^{-\int _{-a}^0\nu (F(t+\xi ))d\xi } \rho (F(t-a))\eta E(t-\tau -a)da]=0, \end{aligned} \right. \end{aligned}$$

or $\frac{d}{dt}D(x_t)=f(x_t)$, where the phase space $X=C([-\tau -T, 0]; R^2)$, $x(t)=(E(t), F(t))$, the neutral operator $D: X\rightarrow R^2$ and the functional $f:X\rightarrow R^2$ are given by

$$\begin{aligned} D(\phi , \psi )=\begin{pmatrix}\phi (0), \psi (0)-\int _0^Te^{-\int _{-a}^0\nu (\psi (\xi ))d\xi } \rho (\psi (-a))\eta \phi (-\tau -a)da\end{pmatrix} \end{aligned}$$

and

$$\begin{aligned} f(\phi , \psi ) =\begin{pmatrix}-\delta \phi (0)+e^{-\int _{-T}^0\nu (\psi (\theta ))d\theta } \rho (\psi (-T))\eta \phi (-\tau -T),0\end{pmatrix} \end{aligned}$$

with $(\phi , \psi )\in X$. The fundamental theory of neutral functional differential equations including the principle of linearization can be found in Hale (1977), Hale and Lunel (1993). See also Barbarossa et al. (2014) and Gourley and Kuang (2009) for neutral equations arising from structured population dynamics in other settings.

## Equilibria and stability of trivial state

The equilibrium of model (4) satisfies the following nonlinear equations

8 $$\begin{aligned} \left\{ \begin{aligned} \delta E&=e^{-\int _{t-T}^t\nu (F)d\theta } \rho (F)\eta E,\\ F&=\int _0^Te^{-\int _{-a}^0\nu (F)d\xi } \rho (F)\eta Eda. \end{aligned} \right. \end{aligned}$$

### Trivial equilibrium and its stability

Clearly, model (4) always has a trivial equilibrium $P_0(0,0)$. We linearize model (4) at the zero equilibrium $P_0$ to obtain

9 $$\begin{aligned} \begin{aligned} \dfrac{dE(t)}{dt}&=-\delta E(t)+\rho (0)\eta e^{-\nu (0)T} E(t-\tau -T). \end{aligned} \end{aligned}$$

The stability of the trivial equilibrium $P_0$ is determined by the following characteristic equation

$$\begin{aligned} \lambda =-\delta +\rho (0)\eta e^{-\nu (0)T}e^{-\lambda (\tau +T)}. \end{aligned}$$

Therefore, the trivial equilibrium $P_0$ is locally asymptotically stable if $\delta >\rho (0)\eta e^{-\nu (0)T}$, and unstable if $\delta <\rho (0)\eta e^{-\nu (0)T}$. This is because of the positive feedback in model (9), and the semi-group generated by this linear delay differential equation is order-preserving, and the stability of the zero solution is the same as that of the following ordinary differential equation

$$\begin{aligned} \dfrac{dE(t)}{dt}=-\delta E(t)+\rho (0)\eta e^{-\nu (0)T} E(t) \end{aligned}$$

by using the monotone dynamical system theory (Smith 1987, 1995). The linearization of the coupled system for the algebraic equation is

$$\begin{aligned} F(t)=\int ^T_0e^{-\nu (0)a}\rho (0)\eta E(t-\tau -a)da, \end{aligned}$$

Therefore, $$F(t)\rightarrow 0$$ as $$t\rightarrow \infty $$.

### Non-trivial equilibria

The nontrivial equilibrium $P_+(E^*,F^*)$ of model (4) satisfies

10 $$\begin{aligned} \left\{ \begin{aligned} \nu (F^*)&=\dfrac{1}{T}\ln \dfrac{\eta \rho (F^*)}{\delta },\\ E^*&=\dfrac{F^*\nu (F^*)}{\eta \rho (F^*)-\delta }, \end{aligned} \right. \end{aligned}$$

where $\delta =\eta \rho (F^*)e^{-\nu (F^*)T}<\eta \rho (F^*)$. Therefore, the existence and multiplicity of nontrivial equilibrium depends on behaviors of tick attachment rate $\rho (F)$ and the host grooming rate $\nu (F)$. We consider several scenarios of the tick-host interface.

#### Constant attaching and grooming

We first consider the simplest case of constant attachment rate and grooming rate, with $\rho (F)=p$ and $\nu (F)=\mu _0$, where $p, \mu _0>0$ are positive constants. Model (4) becomes a linear system as no nonlinearity involves:

11 $$\begin{aligned} \left\{ \begin{aligned} \dfrac{dE(t)}{dt}&=-\delta E(t)+e^{-\mu _0T} p\eta E(t-\tau -T),\\ F(t)&=\int _0^Te^{-\mu _0a} p\eta E(t-\tau -a)da. \end{aligned} \right. \end{aligned}$$

The first equation is a scalar delayed differential equation with a positive delayed feedback. Clearly, the basic reproduction number is $R_0=p\eta e^{-\mu _0T}\delta ^{-1}$, obtained from the multiplication of reproduction and survival probability during the life cycle except the nymphal tick engorgement with sojourn time $\delta ^{-1}$. An application of the Krein-Rutman theorem (see Smith 1987, 1995) shows that solution of *E*(*t*) with a non-trivial non-negative initial value on $[-\tau -T, 0]$ is convergent to 0 or $\infty$ as $t\rightarrow \infty$ when $R_0<1$ and $R_0>1$ respectively. Correspondingly, using the second (integral) equation, we obtain that $F(t)\rightarrow 0$ or $F(t)\rightarrow \infty$ as $t\rightarrow \infty$.

##### Remark 2

Note also that (10) has infinitely many positive equilibria in the critical case when $R_0=1$. Namely, when

12 $$\begin{aligned} \eta p=\delta e^{\mu _0T}, \end{aligned}$$

there are infinitely many positive equilibria $(\frac{\mu _0F^*}{\delta (e^{\mu _0T}-1)},F^*)$, $F^*>0$, in model (11). Then the first differential equation of (11) is reduced into

$$\begin{aligned} \dfrac{dE(t)}{dt}=\delta (E(t-\tau -T)-E(t)). \end{aligned}$$

This type of delay differential equation was studied previously in Haddock-Terjeki (1983) and it has the so-called asymptotic constancy property. That is, $\lim _{t\rightarrow \infty }E(t)=E_c$ exists and is a constant for each given solution. To determine the value $E_c$ for each given solution, we first observe that

$$\begin{aligned} \frac{d}{dt}[E(t)+\delta \int ^t_{t-\tau -T}E(s)ds]=0, \end{aligned}$$

from which it follows that

$$\begin{aligned} E(t)+\delta \int ^t_{t-\tau -T}E(s)ds= E(0)+\delta \int ^0_{-\tau -T}E(s)ds. \end{aligned}$$

Therefore

$$\begin{aligned} E_c=\frac{E(0)+\delta \int ^0_{-\tau -T}E(s)ds}{1+\delta (\tau +T)}. \end{aligned}$$

#### Density-dependent monotone attaching and grooming rates

A more biologically realistic situation is when the attachment rate decreases and grooming rate increases with tick loads on the host. We consider the prototypical case

$$\begin{aligned} \rho (F)=pe^{-F/c}, \quad \nu (F)=\mu F+\mu _0 \end{aligned}$$

for $F\ge 0$, where $c>0$, $\mu >0$ and $\mu _0>0$ are constants. In this case, we can reduce model (4) into

13 $$\begin{aligned} \left\{ \begin{aligned} \dfrac{dE(t)}{dt}&=-\delta E(t)+e^{-\int _{t-T}^t(\mu F(\theta )+\mu _0)d\theta } pe^{-\frac{F(t-T)}{c}}\eta E(t-\tau -T),\\ F(t)&=\int _0^Te^{-\int _{-a}^0(\mu F(t+\xi )+\mu _0)d\xi } pe^{-\frac{F(t-a)}{c}}\eta E(t-\tau -a)da. \end{aligned} \right. \end{aligned}$$

Model (13) has a unique positive equilibrium if $R_0>1$, and no positive equilibrium if $R_0<1$, with $R_0:=p\eta e^{-\mu _0T}\delta ^{-1}$. When $R_0<1$, we can use the following differential inequality

$$\begin{aligned} \frac{dE(t)}{dt}\le -\delta E(t)+p\eta e^{-\mu _0T}E(t-\tau -T) \end{aligned}$$

to conclude that $E(t)\rightarrow 0$ as $t\rightarrow \infty$, and then use the integral inequality

$$\begin{aligned} F(t)\le \int _0^Te^{-\mu _0a}p\eta E(t-\tau -a)da \end{aligned}$$

to conclude that $F(t)\rightarrow 0$ as $t\rightarrow \infty$.

#### Cooperative feeding and density-dependent grooming

Recall that ticks may pool their saliva to enhance the immunomodulatory manipulation of the host organism. The resulted cooperative feeding could increase the cost-benefit ratio of resource extraction from the host relative to per capita investment in tick saliva production. Therefore, in cooperative feeding, the attachment/fixation rate is an increasing function of the feeding tick density on the host. However, since ticks prefer seeking for soft and thin areas of host skin that are well-supplied with blood, there is a maximum capacity to accommodate tick attachments. To describe this cooperative and self-limiting feeding attaching process, we consider the case where the attachment/fixation rate $\rho (x)$ is an initially increasing function that becomes decreasing after the capacity (c) is reached: there exist two constants $p>0$ and $c>0$ satisfying $\rho '(0)=p>0$, $\rho '(F)>0$ for $F\in (0,c)$ and $\rho '(F)<0$ for $F\in (c,+\infty )$. For the sake of simplicity, we use the Ricker function (see Ricker 1975) as a prototypical attachment rate function, i.e.,

14 $$\begin{aligned} \rho (F)=pF e^{-\frac{F}{c}}. \end{aligned}$$

We will couple this cooperative and self-limiting attachment with the density-dependent grooming rate: responding to increasing feeding ticks, the host groom more frequently, resulting in dropping off rate function $\nu (F)$ being an increasing function, i.e.,

$$\begin{aligned} \nu (F)=\mu F+\mu _0. \end{aligned}$$

Then model (4) can be rewritten as

15 $$\begin{aligned} \left\{ \begin{aligned} \dfrac{dE(t)}{dt}&=-\delta E(t)+p\eta e^{-\int _{t-T}^t(\mu F(\theta )+\mu _0)d\theta } F(t-T)e^{-\frac{1}{c}F(t-T)} E(t-\tau -T),\\ F(t)&=p\eta \int _0^Te^{-\int _{-a}^0(\mu F(t+\xi )+\mu _0)d\xi }F(t-a)e^{-\frac{1}{c}F(t-a)} E(t-\tau -a)da. \end{aligned} \right. \end{aligned}$$

To look at a positive equilibrium *F* of model (15), we consider positive solutions of the first equation of (10), namely,

16 $$\begin{aligned} xe^{-(\frac{1}{c}+\mu T)x}=\delta (\eta p)^{-1}e^{\mu _0T}. \end{aligned}$$

The function $g_1(x):=xe^{-(\frac{1}{c}+\mu T)x}$ changes its monotonicity (from increasing to decreasing once), and researches its maximum $e^{-1}(\frac{1}{c}+\mu T)^{-1}$ when $x=(\frac{1}{c}+\mu T)^{-1}$. Hence, we conclude that

##### Theorem 2

The equilibrium structure is determined by $\delta (\eta p)^{-1}e^{\mu _0T}$ and $e^{-1}(\frac{1}{c}+\mu T)^{-1}$. That is,

(i) If $\delta (\eta p)^{-1}e^{\mu _0T}>e^{-1}(\frac{1}{c}+\mu T)^{-1}$, model (15) has no positive equilibrium;
(ii) If $\delta (\eta p)^{-1}e^{\mu _0T}=e^{-1}(\frac{1}{c}+\mu T)^{-1}$, model (15) has only one equilibrium $(E^*, F^*)$, where $F^*=(\frac{1}{c}+\mu T)^{-1}$ and $E^*=\frac{F^*(\mu F^{*}+\mu _0)}{\eta pF^*e^{-F^*/c}-\delta }$;
(iii) If $\delta (\eta p)^{-1}e^{\mu _0T}<e^{-1}(\frac{1}{c}+\mu T)^{-1}$, model (15) has two positive equilibria $(E_-^*,F_-^*)$ and $(E_+^*,F_+^*)$, where $F_{\pm }^*$ are two positive solutions of (16) and the corresponding $E^*_{\pm }$ are given by the second expression of (10) with *F* being replaced by $F^*_\pm$ respectively.

#### Grooming reactive to tick biting

When hosts groom in response to tick biting, we have

$$\begin{aligned} \nu (F)=f(\rho (F)) \end{aligned}$$

with $f(\rho )$ being an increasing function of $\rho \ge 0$. Here, we consider a linear function $f(\rho )=k\rho$. Then model (4) becomes

17 $$\begin{aligned} \left\{ \begin{aligned} \dfrac{dE(t)}{dt}&=-\delta E(t)+e^{-\int _{t-T}^t\nu (F(\theta ))d\theta } \rho (F(t-T))\eta E(t-\tau -T),\\ F(t)&=\int _0^Te^{-\int _{-a}^0\nu (F(t+\xi ))d\xi } \rho (F(t-a))\eta E(t-\tau -a)da. \end{aligned} \right. \end{aligned}$$

The first equation of (10) for the equilibrium state becomes an equation for $\rho$:

$$\begin{aligned} \rho e^{-kT\rho }=\delta \eta ^{-1}. \end{aligned}$$

Let $g_2(\rho ):=\rho e^{-kT\rho }$. Then $g_2'(\rho )=e^{-kT\rho }(1-kT\rho )$, and we obtain the maximum value $(ekT)^{-1}$ of the function $g_2(\rho )$ at $\rho =(kT)^{-1}$. For a corresponding attachment rate function, we use the function

18 $$\begin{aligned} \rho (F)=\dfrac{rF}{1+j^{-2}F^2}, \end{aligned}$$

where $$r>0$$ and $$j>0$$.

The choice of this function is motivated by the Holling type III functional response to describe the improvement of the tick’s searching for an appropriate soft and thin area of the host skin. Note that we have separated the ticks on the hosts into two classes: those questing ticks who have successfully attached to the host but not yet feeding on the host, and those who are feeding, therefore the questing functional response function is $\rho (F)Q$, which would be the saturation function $rF^2/(1+j^{-2}F^2)$ should $Q=F$. We use r for the "attack" rate of the type III functional response (normally denoted by *a*, but this has been reserved for the age-variable in our current study). The parameter *j* is related to the attach rate (*r*) and the handling time (normally denoted by *h*), that is, $j^{-1}=rh$. The function $\rho (F)$ reaches its maximum value *rj*/2 at $F=j$. A simple geometric argument yields

##### Theorem 3

**Fig. 1:**
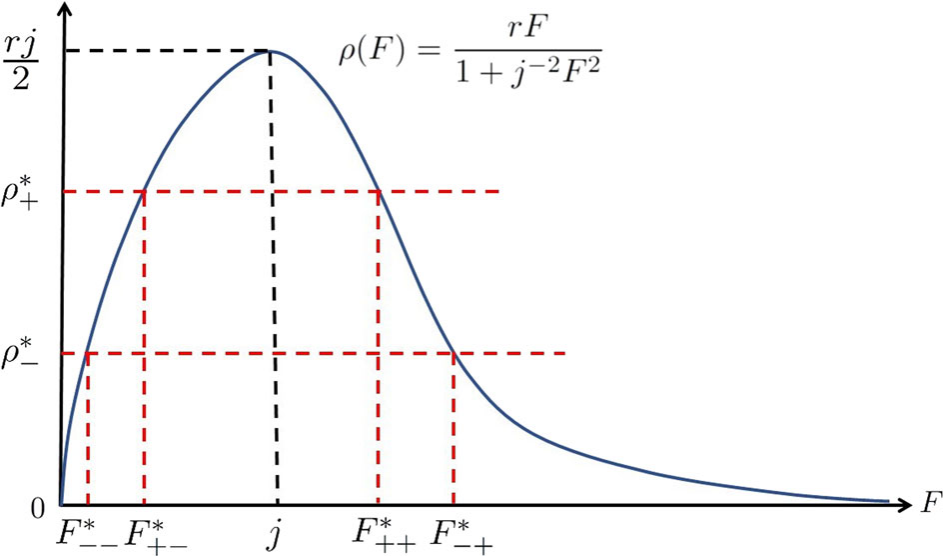
An illustration for the scenario where four positive equilibria may exist when $\delta \eta ^{-1}<(ekT)^{-1}$ and $\rho _+^*< {rj}/{2}$

For the combination of the attachment rate (18) and the linear grooming behaviour $f(\rho )=k\rho$, we have the following results about the equilibrium structure:

(i) When $\delta \eta ^{-1}>(ekT)^{-1}$, there is no positive equilibrium in model (17);
(ii) When $\delta \eta ^{-1}<(ekT)^{-1}$, the equation $g_2(\rho )=\delta \eta ^{-1}$ has two positive solutions $\rho _-^*$ and $\rho _+^*$, which satisfy that $\rho _-^*<(kT)^{-1}<\rho _+^*$. We have the following subcases:
  1. If $\rho _-^*>\frac{rj}{2}$, model (17) has no positive equilibrium;
  2. If $\rho _-^*< \frac{rj}{2}<\rho _+^*$, model (17) has two positive equilibria $(E_{--}^*,F_{--}^*)$ and $(E_{-+}^*, F_{-+}^*)$, where $F_{--}^*$ and $F_{-+}^*$ ($F_{--}^*<j<F_{-+}^*$) are two positive solutions of the equation $\rho (x)=\rho _-^*$; and the corresponding $E^*_{-, \pm }$ are given by the second expression of (10) with *F* being replaced by $F^*_{-\pm }$ respectively;
  3. If $\rho _+^*< \frac{rj}{2}$, model (17) has four positive equilibria $(E_{--}^*,F_{--}^*)$, $(E_{-+}^*, F_{-+}^*)$, $(E_{+-}^*, F_{+-}^*)$ and $(E_{++}^*, F_{++}^*)$, where $F_{-\pm }^*$ and $F_{+\pm }^*$ with $F_{--}^*<F_{+-}^*<j<F_{++}^*<F_{-+}^*$ (see Fig. 1) are positive solutions of the equations $\rho (x)=\rho _-^*$ and $\rho (x)=\rho _+^*$, respectively, and the corresponding $E^*_{\pm , \pm }$ are given by the second expression of (10) with *F* being replaced by $F^*_{\pm \pm }$ respectively.

We illustrate the case (ii3) in Fig. 1, where we see the model has four positive equilibria.

## Multi-stability

### Stability and characteristic equation

We translate the positive equilibrium $P_+$ into the origin by the translation

$$\begin{aligned} \left\{ \begin{aligned}x(t)&=E(t)-E^*,\\y(t)&=F(t)-F^*,\end{aligned}\right. \end{aligned}$$

model (4) becomes

19 $$\begin{aligned} \left\{ \begin{aligned} \dfrac{dx(t)}{dt}&=-\delta (x(t)+E^*)+e^{-\int _{t-T}^t\nu (y(\theta )+F^*)d\theta } \rho \left( y(t-T)+F^*\right) \eta (x(t-\tau -T)+E^*),\\ 0&=\int _0^Te^{-\int _{-a}^0\nu (y(t+\xi )+F^*)d\xi } \rho \left( y(t-a) +F^*\right) \eta (x(t-\tau -T)+E^*)da-y(t)-F^*. \end{aligned} \right. \end{aligned}$$

Model (19) is a delayed differential-algebraic system, also called a degenerate differential system with delay. Linearization of system (19) takes the following form

20 $$\begin{aligned} \left\{ \begin{aligned}\dfrac{dx(t)}{dt}&=-\delta x(t)+\eta e^{-\nu (F^*)T}[\rho (F^*)x(t-\tau -T)+\rho '(F^*)E^*y(t-T)\\&\qquad -\rho (F^*)E^*\nu '(F^*)\int ^t_{t-T}y(\theta )d\theta ],\\ 0&=\int _0^T \eta e^{-\nu (F^*)a}[\rho (F^*)x(t-\tau -a)+\rho '(F^*)E^*y(t-a)\\&\qquad -\rho (F^*)E^*\nu '(F^*)\int ^0_{-a}y(t+\xi )d\xi ] da-y(t). \end{aligned} \right. \end{aligned}$$

Looking for a non-trivial exponential vector function as a solution

21 $$\begin{aligned} \left\{ \begin{aligned}x(t)=e^{\lambda t}x_0,\\ y(t)=e^{\lambda t}y_0, \end{aligned} \right. \end{aligned}$$

where $(x_0, y_0)^T\ne 0$, we obtain

22 $$\begin{aligned} \begin{bmatrix}~\begin{pmatrix}\lambda &{} 0\\ 0 &{} 0\end{pmatrix}-\begin{pmatrix}a_{11}(\lambda ) &{} a_{12}(\lambda )\\ a_{21}(\lambda ) &{} a_{22}(\lambda )\end{pmatrix}~\end{bmatrix}\begin{pmatrix}x_0\\ y_0\end{pmatrix}=0, \end{aligned}$$

where

$$\begin{aligned} \begin{aligned} a_{11}(\lambda )=&-\delta +\eta e^{-\nu (F^*)T}\rho (F^*)e^{-\lambda (\tau +T)},\\ a_{12}(\lambda )=&\eta e^{-\nu (F^*)T}E^*[\rho '(F^*)e^{-\lambda T}-\frac{\rho (F^*)\nu '(F^*)}{\lambda }(1-e^{-\lambda T})],\\ a_{21}(\lambda )=&\frac{\eta \rho (F^*)e^{-\lambda \tau }}{\nu (F^*)+\lambda }(1-e^{-(\nu (F^*)+\lambda )T}),\\ a_{22}(\lambda )=&\frac{\eta E^*}{\nu (F^*)+\lambda }\left( \rho '(F^*)+\frac{\rho (F^*)\nu '(F^*)}{\lambda }\right) (1-e^{-(\nu (F^*)+\lambda )T})\\&-\frac{\eta \rho (F^*)E^*\nu '(F^*)}{\lambda \nu (F^*)}(1-e^{-\nu (F^*)T})-1. \end{aligned} \end{aligned}$$

In order for $(x_0,y_0)^T$ to be a nonzero vector, the determinant of the coefficient matrix of the system (22) should be zero, i.e., the characteristic equation of (20) takes the following form

23 $$\begin{aligned} f(\lambda )=\begin{vmatrix}\begin{array}{cc}\lambda -a_{11}(\lambda ) &{} -a_{12}(\lambda )\\ -a_{21}(\lambda ) &{} -a_{22}(\lambda )\end{array}\end{vmatrix}=0. \end{aligned}$$

It is highly nontrivial to describe the distribution of zeros of this characteristic equation in the general case. In what follows, we take advantage of the fact that the feeding duration *T* is relatively small (a few days) comparing with the tick life cycle in the natural world that can be as long as 3 years.

### Bi-stability: cooperative feeding and density-dependent grooming

In this section, we use feeding duration $T=\epsilon >0$ as a parameter and focus on the stability of model (15) at positive equilibria $(E^*_{-}(\epsilon ),F^*_{-}(\epsilon ))$ and $(E^*_{+}(\epsilon ),F^*_{+}(\epsilon ))$ obtained in Theorem 2.

We start with the simplest case where $\epsilon =0$. If $c/e>\delta /(\eta p)$, this special case reduces the positive equilibria of model (15) into $(E^0_{-},F^0_{-})$ and $(E^0_{+},F^0_{+})$, where $F^0_{-}$, $F^0_{+}$ ($F^0_{-}<c<F^0_{+}$) are the solutions of the following equation

$$\begin{aligned} Fe^{-\frac{F}{c}}=\delta (\eta p)^{-1}, \end{aligned}$$

from which we obtain the corresponding *E*-coordinate:

$$\begin{aligned} E^0_{-}=\frac{F^0_{-}\nu (F^0_{-})}{\eta \rho (F^0_{-})-\delta }; \;\; E^0_{+}=\frac{F^0_{+}\nu (F^0_{+})}{\eta \rho (F^0_{+})-\delta }. \end{aligned}$$

Using the implicit function theorem, when $T=\epsilon >0$, it can be seen that model (15) has two positive equilibria $(E^*_{-}(\epsilon ),F^*_{-}(\epsilon ))$ and $(E^*_{+}(\epsilon ),F^*_{+}(\epsilon ))$, where $F^*_{-}(\epsilon )$, $F^*_{+}(\epsilon )$ satisfying $F^*_{-}(\epsilon )<c<F^*_{+}(\epsilon )$ are the two positive solutions of the following equation

$$\begin{aligned} Fe^{-(\frac{1}{c}+\mu \epsilon )F}=\delta (\eta p)^{-1}e^{\mu _0\epsilon }, \end{aligned}$$

and

$$\begin{aligned} E^*_{-}(\epsilon )=\dfrac{F^*_{-}(\epsilon )\nu (F^*_{-}(\epsilon ))}{\eta \rho (F^*_{-}(\epsilon ))-\delta },\quad E^*_{+}(\epsilon )=\dfrac{F^*_{+}(\epsilon )\nu (F^*_{+}(\epsilon ))}{\eta \rho (F^*_{+}(\epsilon ))-\delta }. \end{aligned}$$

Now, considering $T=\epsilon$ as a variable parameter, we focus on searching for the characteristic eigenvalue of linearized system (20) with respect to $\epsilon$. As the equilibrium $E(\epsilon )$ has a singularity as $\epsilon \rightarrow 0$, the characteristic equation has a singularity at $\epsilon =0$ so we need to single out this singularity. Define

$$\begin{aligned} G(\lambda , \epsilon )=\epsilon [(\lambda (\epsilon )-a_{11}(\lambda ,\epsilon ))a_{22}(\lambda ,\epsilon ) +a_{12}(\lambda ,\epsilon )a_{21}(\lambda ,\epsilon )] \end{aligned}$$

with $G(\lambda ,0)=\displaystyle \lim _{\epsilon \rightarrow 0^+} G(\lambda ,\epsilon )$ which is given by

24 $$\begin{aligned} \begin{pmatrix}\lambda -a_{11}(\lambda ,0)\end{pmatrix}\cdot \displaystyle \lim _{\epsilon \rightarrow 0} \begin{pmatrix}\epsilon a_{22}(\lambda ,\epsilon )\end{pmatrix}+a_{21}(\lambda ,0)\cdot \displaystyle \lim _{\epsilon \rightarrow 0}\begin{pmatrix}\epsilon a_{12}(\lambda ,\epsilon )\end{pmatrix} =0. \end{aligned}$$

Therefore, we consider $G(\lambda ,\epsilon )=0$ as the characteristic equation of system (19). In what follows, we will calculate the series expansion of $G(\lambda ,\epsilon )=0$ with respect to $\epsilon$.

It is clear to see that

$$\begin{aligned} \begin{aligned} \epsilon E_{\pm }^*(\epsilon )&=\dfrac{F_{\pm }^*(\epsilon )\nu (F_{\pm }^*(\epsilon ))\epsilon }{\eta \rho (F_{\pm }^*(\epsilon ))-\delta }=\dfrac{F_{\pm }^*(\epsilon )\nu (F_{\pm }^*(\epsilon ))\epsilon }{\delta e^{\nu (F_{\pm }^*(\epsilon ))\epsilon }-\delta }=\dfrac{F_{\pm }^*(\epsilon )\nu (F_{\pm }^*(\epsilon ))\epsilon }{\delta \nu (F_{\pm }^*(\epsilon ))\epsilon +o(\epsilon )}\\ {}&\rightarrow \frac{F_{\pm }^0}{\delta },~~ \text {as} ~ \epsilon \rightarrow 0. \end{aligned} \end{aligned}$$

Thus,

25 $$\begin{aligned} \epsilon a_{12}(\lambda , \epsilon )\rightarrow \eta \delta ^{-1}\rho '(F_{\pm }^0)F_{\pm }^0 \quad \text {and} \quad \epsilon a_{22}(\lambda ,\epsilon )\rightarrow 0, ~ \text {as}~ \epsilon \rightarrow 0. \end{aligned}$$

Substituting (25) into (24), we can get $G(\lambda (0),0)=0$. Then it can be seen that $G(\lambda , \epsilon )$ is a $C^1$-smooth function defined for $\epsilon$ in a neighbourhood of zero and $G(\lambda , \epsilon )=0$ becomes the characteristic equation of (15) when $\epsilon >0$.

We now consider the real part of a zero of the modified but equivalent characteristic equation $G(\lambda , \epsilon )=0$, at positive equilibria $(F_{-}^*(\epsilon ),E_{-}^*(\epsilon ))$ and $(F_{+}^*(\epsilon ),E_{+}^*(\epsilon ))$, respectively.

Suppose that the series expansions of $F(\epsilon )$ and $\epsilon E(\epsilon )$ with the following forms

26 $$\begin{aligned} \begin{aligned} F(\epsilon )=F_0+F_1\epsilon +o(\epsilon ),\\ \epsilon E(\epsilon )=\frac{F_0}{\delta }+E_1\epsilon +o(\epsilon ), \end{aligned} \end{aligned}$$

where $o(\epsilon )$ represents higher order terms. From the expressions of $\rho (F)$ and $\nu (F)$, we obtain the following series expansions

27 $$\begin{aligned} \rho (F)&=p(F_0+F_1\epsilon +o(\epsilon ))e^{-\frac{1}{c}(F_0+F_1\epsilon +o(\epsilon ))}\nonumber \\&=\rho _0+\rho _1\epsilon +0(\epsilon ),\nonumber \\ \nu (F)&=\mu (F_0+F_1\epsilon +o(\epsilon ))+\mu _0\nonumber \\&=\nu _0+\nu _1\epsilon +o(\epsilon ),\nonumber \\ \rho '(F)&=p(1-\frac{F}{c})e^{-\frac{F}{c}}=p(1-\frac{F}{c})(1-\frac{F}{c}+\frac{F^2}{2!c^2}+o(F^2))\nonumber \\&={\tilde{\rho }}_0+{\tilde{\rho }}_1\epsilon +o(\epsilon ),\nonumber \\ \nu '(F)&=\mu , \end{aligned}$$

where $\rho _0=pF_0e^{-\frac{F_0}{c}}$, $\rho _1=pF_1(1-\frac{F_0}{c})e^{-\frac{F_0}{c}}$, $\nu _0=\mu _0+\mu F_0$, $\nu _1=\mu F_1$, ${\tilde{\rho }}_0=pe^{-\frac{F_0}{c}}(1-\frac{F_0}{c})$, ${\tilde{\rho }}_1=-\frac{p}{c}F_1e^{-\frac{F_0}{c}}(2-\frac{F_0}{c})$. Combining the above series expansions, the fist equation of (10) can be rewritten as

$$\begin{aligned} \begin{aligned} \delta&=\eta \rho (F)e^{-\nu (F)\epsilon }\\&=\eta (\rho _0+\rho _1\epsilon +o(\epsilon ))\cdot [1-(\nu _0+\nu _1\epsilon )\epsilon +\dfrac{1}{2!}\nu _0^2\epsilon ^2+o(\epsilon ^2)], \end{aligned} \end{aligned}$$

which leads to

28 $$\begin{aligned} \delta =\eta \rho _0~~ \text {and}~~ \rho _1=\nu _0\rho _0. \end{aligned}$$

Based on the relationship in (28), we have

$$\begin{aligned} F_1=\dfrac{(\mu _0+\mu F_0)F_0}{1-\frac{F_0}{c}}. \end{aligned}$$

Using the second equation of (8), we have

29 $$\begin{aligned} F=\eta \dfrac{\rho (F)}{\nu (F)} E(1-e^{-\nu (F)\epsilon })=\eta \rho (F)\epsilon E[1-\frac{1}{2!}\nu (F)\epsilon +\frac{1}{3!}\nu ^2(F)\epsilon ^2+o(\epsilon ^2)]. \end{aligned}$$

Substituting the series expansions of $F(\epsilon )$, $\epsilon E(\epsilon )$, $\rho (F)$, $\nu (F)$ into (29), we yield

$$\begin{aligned} \begin{aligned} \delta&=\eta pF_0e^{-\frac{F_0}{c}},\\ F_1&=\eta (\rho _0E_1+\frac{1}{\delta }\rho _1F_0-\dfrac{1}{2\delta }\rho _0\nu _0F_0). \end{aligned} \end{aligned}$$

From all coefficients in (27) and the relationship in (28), we have

$$\begin{aligned} E_1=\dfrac{1}{\delta }(\mu F_0+\mu _0)F_0(\dfrac{c}{c-F_0}-\dfrac{1}{2}). \end{aligned}$$

Thus, the series expansions in (26) can be expressed into

$$\begin{aligned} \begin{aligned} F(\epsilon )&=F_0+\dfrac{(\mu _0+\mu F_0)F_0}{1-\frac{F_0}{c}}\epsilon +o(\epsilon ),\\ \epsilon E(\epsilon )&=\dfrac{F_0}{\delta }+\dfrac{1}{\delta }(\mu F_0+\mu _0)F_0(\dfrac{c}{c-F_0}-\dfrac{1}{2})\epsilon +o(\epsilon ). \end{aligned} \end{aligned}$$

Assume that the series expansion of eigenvalue satisfying $G(\lambda ,\epsilon )=0$ is given by

$$\begin{aligned} \lambda (\epsilon )=\lambda _0+\lambda _1\epsilon +o(\epsilon ). \end{aligned}$$

Based on the expressions of $a_{ij}$ in (22), we can calculate their series expansions as follows

$$\begin{aligned} \begin{aligned} a_{11}(\epsilon )&=-\delta +\eta e^{-(\nu _0+\nu _1\epsilon +o(\epsilon ))\epsilon }(\rho _0 +\rho _1\epsilon +o(\epsilon ))e^{-(\tau +\epsilon )(\lambda _0+\lambda _1\epsilon +o(\epsilon ))}\\&=\delta (-1+e^{-\lambda _0\tau })-\delta e^{-\lambda _0\tau }(\lambda _0+\lambda _1\tau )\epsilon +o(\epsilon ),\\ a_{21}(\epsilon )&=\dfrac{\eta (\rho _0+\rho _1\epsilon +o(\epsilon ))e^{-(\lambda _0 +\lambda _1\epsilon +o(\epsilon ))\tau }}{(\nu _0+\lambda _0)+(\nu _1+\lambda _1)\epsilon +o(\epsilon )}(1-e^{-(\nu _0+\lambda _0+(\nu _1+\lambda _1)\epsilon +o(\epsilon ))\epsilon })\\&=\eta \rho _0e^{-\lambda _0\tau }\epsilon +o(\epsilon ),\\ \epsilon a_{12}(\epsilon )&=\eta e^{-(\nu _0+\nu _1\epsilon +o(\epsilon ))\epsilon } (E_0+E_1\epsilon +o(\epsilon ))[({\tilde{\rho }}_0+{\tilde{\rho }}_1\epsilon +o(\epsilon ))e^{-(\lambda _0+\lambda _1\epsilon +o(\epsilon ))\epsilon }\\&\quad -\frac{(\rho _0+\rho _1\epsilon +o(\epsilon ))\mu }{\lambda _0+\lambda _1\epsilon +o(\epsilon )}(1-e^{-(\lambda _0+\lambda _1\epsilon +o(\epsilon ))\epsilon })]\\&=\eta E_0{\tilde{\rho }}_0+\eta [-\nu _0E_0{\tilde{\rho }}_0+E_1{\tilde{\rho }}_0 +E_0({\tilde{\rho }}_1-{\tilde{\rho }}_0\lambda _0-\rho _0\mu )]\epsilon +o(\epsilon ),\\ \epsilon a_{22}(\epsilon )&=\dfrac{\eta (E_0+E_1\epsilon +o(\epsilon ))}{\nu _0 +\lambda _0+(\nu _1+\lambda _1)\epsilon +o(\epsilon )}({\tilde{\rho }}_0+{\tilde{\rho }}_1\epsilon +o(\epsilon )\\&\quad +\dfrac{(\rho _0+\rho _1\epsilon +o(\epsilon ))\mu }{\lambda _0+\lambda _1\epsilon +o(\epsilon )})(1-e^{-(\nu _0+\lambda _0+(\nu _1+\lambda _1)\epsilon +o(\epsilon ))\epsilon })\\&\quad -\dfrac{\eta \mu (\rho _0+\rho _1\epsilon +o(\epsilon ))(E_0+E_1\epsilon +o(\epsilon ))}{(\nu _0+\nu _1\epsilon +o(\epsilon ))(\lambda _0+\lambda _1\epsilon +o(\epsilon ))} (1-e^{-(\nu _0+\nu _1\epsilon +o(\epsilon ))\epsilon })-\epsilon \\&=(-1+\eta E_0{\tilde{\rho }}_0)\epsilon +o(\epsilon ). \end{aligned} \end{aligned}$$

The characteristic polynomial $G(\lambda ,\epsilon )$ becomes

$$\begin{aligned} G(\lambda ,\epsilon )=[(-1+\eta E_0{\tilde{\rho }}_0 )(\lambda _0-\delta (-1+e^{-\lambda _0\tau }))+\eta E_0{\tilde{\rho }}_0\eta \rho _0e^{-\lambda _0\tau }]\epsilon +o(\epsilon ). \end{aligned}$$

From the characteristic equation $G(\lambda ,\epsilon )=0$, it follows that

30 $$\begin{aligned} (-1+\eta E_0{\tilde{\rho }}_0 )(\lambda _0-\delta (-1+e^{-\lambda _0\tau }))+\eta E_0{\tilde{\rho }}_0\eta \rho _0e^{-\lambda _0\tau }=0. \end{aligned}$$

Using the expressions in (27) and (28), we can simplify (30) into the following form

$$\begin{aligned} -\dfrac{F_0}{c}\lambda _0+\delta e^{-\lambda _0\tau }=\dfrac{F_0}{c}\delta . \end{aligned}$$

The stability of the two positive equilibria $(F_-^*(\epsilon ),E_-^*(\epsilon ))$ and $(F_+^*(\epsilon ),E_+^*(\epsilon ))$ depends on the sign of the first non-zero term $\lambda _0$. In the series expansion of $F(\epsilon )$ in (26), we know that the first term $F_0$ is equal to the equilibrium $F_-^*(\epsilon )$ or $F_+^*(\epsilon )$. Since $F_-^*(\epsilon )<c<F_+^*(\epsilon )$, it follows that

$$\begin{aligned} \left\{ \begin{aligned}&\text {Sign}(\lambda _0)>0, \quad \text {at the positive equilibrium} ~ (F_-^*(\epsilon ),E_-^*(\epsilon )),\\&\text {Sign}(\lambda _0)<0, \quad \text {at the positive equilibrium} ~ (F_+^*(\epsilon ),E_+^*(\epsilon )). \end{aligned} \right. \end{aligned}$$

Then we have the following result:

#### Theorem 4

Assume $\eta p>\delta ec^{-1}$. When the average feeding duration of nymphal ticks $T=\epsilon >0$ is small, bi-stability occurs in model (15) in the cooperative feeding and density-dependent grooming case considered in Theorem 1: the trivial equilibrium $P_0$ and the positive equilibrium $(F_+^*(\epsilon ),E_+^*(\epsilon ))$ are both locally asymptotically stable, and the positive equilibrium $(F_-^*(\epsilon ),E_-^*(\epsilon ))$ is unstable.

We note that in the case considered in Theorem 3, the trivial equilibrium is asymptotically stable since $\rho (0)=0$.

### Quadri-stability with grooming reactive to tick biting

We consider the case that $k =O(\epsilon ^{-1})$. Consider the expansion $k\epsilon$ as follows

$$\begin{aligned} k\epsilon =k_0+k_1\epsilon +o(\epsilon ). \end{aligned}$$

Then the first equation of (10) becomes

31 $$\begin{aligned} \delta \eta ^{-1}=e^{-(k_0+k_1\epsilon +o(\epsilon ))\rho }\rho . \end{aligned}$$

For $\epsilon =0$, Equation (31) has two solutions $\rho _{-0}$ and $\rho _{+0}$ with $0<\rho _{-0}<k_0^{-1}<\rho _{+0}$ if $(ek_0)^{-1}>\delta \eta ^{-1}$. When $\rho _{+0}<rj/2$, by solving the quadratic equation (18), we obtain four positive solutions $F_{--}^0<F_{+-}^0<j<F_{++}^0<F_{-+}^0$ as follows:

$$\begin{aligned} F_{--}^0= & {} \dfrac{\frac{r}{\rho _{-0}}-\sqrt{(\frac{r}{\rho _{-0}})^2-4j^{-2}}}{2j^{-2}},\\ F_{-+}^0= & {} \dfrac{\frac{r}{\rho _{-0}}+\sqrt{(\frac{r}{\rho _{-0}})^2-4j^{-2}}}{2j^{-2}},\\ F_{+-}^0= & {} \dfrac{\frac{r}{\rho _{+0}}-\sqrt{(\frac{r}{\rho _{+0}})^2-4j^{-2}}}{2j^{-2}},\\ F_{++}^0= & {} \dfrac{\frac{r}{\rho _{+0}}+\sqrt{(\frac{r}{\rho _{+0}})^2-4j^{-2}}}{2j^{-2}}. \end{aligned}$$

We now look for $F_{--}(\epsilon )<F_{+-}(\epsilon )<j<F_{++}(\epsilon )<F_{-+}(\epsilon )$ such that

$$\begin{aligned} \delta \eta ^{-1}=e^{(k_0+k_1\epsilon +o(\epsilon ))\rho (F)}\rho (F), \end{aligned}$$

and

$$\begin{aligned} F_{--}(0)=F_{--}^0, ~~ F_{+-}(0)=F_{+-}^0, ~~ F_{++}(0)=F_{++}^0, ~~ F_{-+}(0)=F_{-+}^0. \end{aligned}$$

For the four positive equilibria $(F_{--}(\epsilon ), E_{--}(\epsilon ))$, $(F_{+-}(\epsilon ), E_{+-}(\epsilon ))$, $(F_{++}(\epsilon ), E_{++}(\epsilon ))$ and $(F_{-+}(\epsilon ), E_{-+}(\epsilon ))$ when $\epsilon >0$, we suppose that the series expansions are expressed as

32 $$\begin{aligned} \begin{aligned} F&=F_0+F_1\epsilon +o(\epsilon ),\\ \rho (F)&=\rho _0+\rho _1\epsilon +o(\epsilon ),\\ \rho '(F)&={\tilde{\rho }}_0+{\tilde{\rho }}_1\epsilon +o(\epsilon ),\\ \epsilon E&=E_0+E_1\epsilon +o(\epsilon ),\\ \lambda&=\lambda _0+\lambda _1\epsilon +o(\epsilon ), \end{aligned} \end{aligned}$$

where $F_0$ denotes the positive solutions $F_{--}^0$, $F_{+-}^0$, $F_{++}^0$, $F_{-+}^0$, $\rho _0$ represents $\rho _{-0}$ and $\rho _{+0}$.

Using the attachment rate function (18), we get

33 $$\begin{aligned} \rho _{0}=\frac{rF_{0}}{1+j^{-2}F_{0}^2},~~ \rho _1=\dfrac{rF_1(1-j^{-2}F_0^2)}{(1+j^{-2}F_0^2)^2}, \end{aligned}$$

and

$$\begin{aligned} {\tilde{\rho }}_0=\frac{r(1-j^{-2}F_0^2)}{(1+j^{-2}F_0^2)^2}, ~~ {\tilde{\rho }}_1=-\frac{2rj^{-2}F_0F_1(3-j^{-2}F_0^2)}{(1+j^{-2}F_0^2)^3}. \end{aligned}$$

As we did in last subsection, we now use the method of series expansions to look for the signs of the real parts of the corresponding characteristic equation. In what follows, we drop all the subscripts (“−” and “$+$”) for simplicity.

Let

$$\begin{aligned} H(\lambda ,\epsilon )=\epsilon [(\lambda (\epsilon )-a_{11}(\lambda ,\epsilon ))a_{22} (\lambda ,\epsilon )+a_{12}(\lambda ,\epsilon )a_{21}(\lambda ,\epsilon )]. \end{aligned}$$

We can calculate that

$$\begin{aligned} \begin{aligned} \epsilon E(\epsilon )&=\epsilon \dfrac{F\nu (F)}{\eta \rho (F)-\delta }=\epsilon \dfrac{F\nu (F)}{\eta \rho (F)-\eta e^{-\nu (F)\epsilon }\rho (F)}=\dfrac{F(k\epsilon )}{\eta (1-e^{-k\epsilon \rho })}\\&\rightarrow \dfrac{F_0k_0}{\eta (1-e^{-k_0\rho _0})}, ~~ \text {as}~ \epsilon \rightarrow 0, \end{aligned} \end{aligned}$$

Thus, we have

$$\begin{aligned} E_0=\dfrac{F_0k_0}{\eta (1-e^{-k_0\rho _0})}. \end{aligned}$$

When $\epsilon \rightarrow 0$, we have

$$\begin{aligned} \begin{aligned} \epsilon a_{12}(\lambda ,\epsilon )&=\eta e^{-k\epsilon \rho }\epsilon E[e^{-\lambda \epsilon }-\rho (F)k\epsilon (1-\frac{1}{2!}\lambda \epsilon +o(\epsilon ))]\rho '(F)\\&\rightarrow \dfrac{e^{-k_0\rho _0}F_0k_0}{1-e^{-k_0\rho _0}}{\tilde{\rho }}_0(1-\rho _0k_0),\\ a_{21}(\lambda ,\epsilon )&=\dfrac{\eta \rho (F)e^{-\lambda \tau }\epsilon }{(k\rho (F)+\lambda ) \epsilon }[1-e^{-(k\rho +\lambda )\epsilon }]\rightarrow 0,\\ \epsilon a_{22}(\lambda ,\epsilon )&=\dfrac{\eta E\epsilon \rho '(F)}{\lambda } (1-e^{-(k\rho +\lambda )\epsilon })-\dfrac{\eta \epsilon E\rho '(F)}{\lambda }(1-e^{-k\rho \epsilon })-\epsilon \rightarrow 0,\\ \epsilon a_{11}(\lambda ,\epsilon )&=-\delta +\eta e^{-k\rho \epsilon } \rho (F)e^{-\lambda (\tau +\epsilon )}\rightarrow -\delta +\eta e^{-(k_0\rho _0+\lambda _0\tau )}\rho _0. \end{aligned} \end{aligned}$$

Substituting these limit values above into $H(\lambda , \epsilon )$, we have $H(\lambda (0),0)=0$. Since $H(\lambda ,\epsilon )$ is a $C^1$-smooth function defined for $\epsilon$ in a neighbourhood of zero, $H(\lambda ,\epsilon )=0$ is equivalent to the characteristic equation of (17) when $\epsilon >0$.

In what follows, we will explore the solutions of $H(\lambda ,\epsilon )=0$ at each equilibrium $(F_{--}(\epsilon ), E_{--}(\epsilon ))$, $(F_{+-}(\epsilon ), E_{+-}(\epsilon ))$, $(F_{++}(\epsilon ), E_{++}(\epsilon ))$ and $(F_{-+}(\epsilon ), E_{-+}(\epsilon ))$.

The second equation of (10) gives

34 $$\begin{aligned} F=\dfrac{1}{\nu (F)}\rho (F)\eta E(1-e^{-\nu (F)\epsilon })=\rho (F)\eta \epsilon E[1-\dfrac{1}{2!}\nu (F)\epsilon +\dfrac{1}{3!}\nu ^2(F)\epsilon ^2+o(\epsilon ^2)]. \end{aligned}$$

Combining with (32), we have

35 $$\begin{aligned} F_0=&\dfrac{\eta E_0}{k_0}(1-e^{-k_0\rho _0}), \end{aligned}$$

36 $$\begin{aligned} F_1=&\dfrac{\eta (\rho _1 E_0+\rho _0E_1)}{k_0\rho _0}(1-e^{-k_0\rho _0}) +\dfrac{\eta \rho _0E_0(\rho _0k_1+\rho _1k_0)}{k_0^2\rho _0^2} \end{aligned}$$

37 $$\begin{aligned}&\cdot (k_0\rho _0e^{-k_0\rho _0}+e^{-k_0\rho _0}-1). \end{aligned}$$

According to the first equation of (10), we can obtain

$$\begin{aligned} \delta =\eta e^{-\nu (F)\epsilon }\rho (F)=\eta e^{-(k_0+k_1\epsilon +o(\epsilon ))(\rho _0+\rho _1\epsilon +o(\epsilon ))}(\rho _0+\rho _1\epsilon +o(\epsilon )), \end{aligned}$$

which leads to

38 $$\begin{aligned} \delta&=\eta e^{-k_0\rho _0}\rho _0, \end{aligned}$$

39 $$\begin{aligned} \rho _1&=\rho _0(k_0\rho _1+k_1\rho _0). \end{aligned}$$

Substituting (33) into (39), we have

40 $$\begin{aligned} F_1=\dfrac{rk_1F_0^2}{(1-j^{-2}F_0^2)(1-\dfrac{rF_0k_0}{1+j^{-2}F_0^2})}. \end{aligned}$$

Then $\rho _1$ in (33) can be rewritten as

$$\begin{aligned} \begin{aligned} \rho _1&=\dfrac{r(1-j^{-2}F_0^2)}{(1+j^{-2}F_0^2)^2}\cdot \dfrac{rk_1F_0^2}{(1-j^{-2}F_0^2)(1-\dfrac{rF_0k_0}{1+j^{-2}F_0^2})}\\&=\dfrac{\rho _0 rF_0k_1}{1+j^{-2}F_0^2-rF_0k_0}. \end{aligned} \end{aligned}$$

From (36), it is easy to get

$$\begin{aligned} \begin{aligned} E_1=&\dfrac{k_0}{\eta (1-e^{-k_0\rho _0})}[F_1-\dfrac{\rho _1\eta E_0}{k_0\rho _0}(1-e^{-k_0\rho _0})-\dfrac{\eta E_0}{k_0^2\rho _0}(k_1\rho _0+k_0\rho _1)\\ {}&\cdot (k_0\rho _0e^{-k_0\rho _0}+e^{-k_0\rho _0}-1)]. \end{aligned} \end{aligned}$$

Based on the obtained expressions of $a_{ij}$ in (22), we get

$$\begin{aligned} \begin{aligned} a_{11}(\lambda , \epsilon )&=-\delta +\eta e^{-(k_0+k_1\epsilon +o(\epsilon ))(\rho _0 +\rho _1\epsilon +o(\epsilon ))}(\rho _0+\rho _1\epsilon +o(\epsilon )) e^{-(\lambda _0+\lambda _1\epsilon +o(\epsilon ))(\tau +\epsilon )}\\&=-\delta +\delta e^{-\lambda _0\tau }+\eta e^{-(k_0\rho _0+\lambda _0\tau )} [\rho _1-\rho _0(k_0\rho _1+k_1\rho _0+\lambda _0+\lambda _1\tau )]\epsilon +o(\epsilon ),\\ a_{21}(\lambda , \epsilon )&=\dfrac{\eta \rho (F)e^{-\lambda \tau }}{\nu (F)+\lambda } \begin{pmatrix}1-[1-(\nu (F)+\lambda )\epsilon +\dfrac{1}{2!}(\nu (F)+\lambda )^2\epsilon ^2+o(\epsilon ^2)]\end{pmatrix}\\&=\dfrac{\eta }{k_0}e^{-\lambda _0\tau }(1-e^{-k_0\rho _0})\epsilon +o(\epsilon ),\\ \epsilon a_{12}(\lambda , \epsilon )&=\eta e^{-\nu (F)\epsilon }\epsilon E\rho '(F) [e^{-\lambda \epsilon }-\dfrac{k\rho }{\lambda }(1-e^{-\lambda \epsilon })]\\&=\eta e^{-k_0\rho _0}E_0{\tilde{\rho }}_0(1-k_0\rho _0)+\eta e^{-k_0\rho _0} [E_1{\tilde{\rho }}_0(1-k_0\rho _0)+E_0{\tilde{\rho }}_1(1-k_0\rho _0)\\&\quad -(k_0\rho _1+k_1\rho _0)E_0{\tilde{\rho }}_0(2-k_0\rho _0) -\lambda _0E_0{\tilde{\rho }}_0(1-\dfrac{1}{2}k_0\rho _0)]\epsilon +o(\epsilon ),\\ \epsilon a_{22}(\lambda , \epsilon )&=\dfrac{\eta \epsilon E}{\nu (F)+\lambda }\rho '(F) (1+\dfrac{k\rho }{\lambda })(1-e^{-(\nu (F)+\lambda )\epsilon })-\dfrac{\eta \epsilon E\rho '(F)}{\lambda }(1-e^{-k\rho \epsilon })-\epsilon \\&=(-1+\eta E_0{\tilde{\rho }}_0e^{-k_0\rho _0})\epsilon +o(\epsilon ). \end{aligned} \end{aligned}$$

Substituting $a_{11}(\lambda , \epsilon )$, $a_{21}(\lambda , \epsilon )$, $\epsilon a_{12}(\lambda , \epsilon )$ and $\epsilon a_{22}(\lambda , \epsilon )$ into the characteristic equation $H(\lambda ,\epsilon )=0$, we have

$$\begin{aligned}{} & {} {[}(-1+\eta E_0{\tilde{\rho }}_0e^{-k_0\rho _0})(\lambda _0+\delta -\delta e^{-\lambda _0\tau })\\{} & {} \quad +\dfrac{\eta ^2E_0{\tilde{\rho }}_0}{k_0}e^{-k_0\rho _0 -\lambda _0\tau }(1-k_0\rho _0)(1-e^{-k_0\rho _0})]\epsilon +o(\epsilon ) =0. \end{aligned}$$

Therefore,

41 $$\begin{aligned} \lambda _0+\delta -\delta e^{-\lambda _0\tau }+Ae^{-\lambda _0\tau }=0, \end{aligned}$$

where we have

$$\begin{aligned} A=\dfrac{\eta ^2E_0{\tilde{\rho }}_0e^{-k_0\rho _0}(1-k_0\rho _0) (1-e^{-k_0\rho _0})}{k_0(\eta E_0{\tilde{\rho }}_0e^{-k_0\rho _0}-1)}. \end{aligned}$$

It is clear that (41) is the characteristic equation for the following system

42 $$\begin{aligned} \dot{x}=-\delta x(t)+(\delta -A)x(t-\tau ), \end{aligned}$$

which is a delay differential equation with positive feedback if $\delta >A$. The stability of (42) is equivalent to the stability of the zero solution for the following ordinary differential equation (see Smith 1987)

$$\begin{aligned} \dot{x}=-\delta x(t)+(\delta -A)x(t)=-Ax(t). \end{aligned}$$

Then we have the following result:

#### Theorem 5

Assume that $\delta >A$. The stability of equilibria

$$\begin{aligned} (F_{--}(\epsilon ), E_{--}(\epsilon )), (F_{+-}(\epsilon ), E_{+-}(\epsilon )), (F_{++}(\epsilon ), E_{++}(\epsilon )), (F_{-+}(\epsilon ), E_{-+}(\epsilon )) \end{aligned}$$

are determined by the signs of A, respectively, i.e., the equilibrium for model (17) is stable (unstable) if and only if $A>0$ ($A<0$).

Now we assume that

43 $$\begin{aligned} \eta E_0{\tilde{\rho }}_0 e^{-k_0\rho _0}<1. \end{aligned}$$

Since $\delta =\eta \rho _0 e^{-k_0\rho _0}$, we can reduce the condition $\delta >A$ into

$$\begin{aligned} -\rho _0 k_0<\eta E_0{\tilde{\rho }}_0(1-k_0\rho _0-e^{-k_0\rho _0}). \end{aligned}$$

Let $q(x)=1-x-e^{-x}$. Its derivative $q'(x)=e^{-x}-1<0$ for all $x>0$. Therefore, *q*(*x*) is a decreasing function for $x>0$. Note that $q(0)=0$, so $q(x)<0$ for all $x>0$, i.e., $1-k_0\rho _0-e^{-k_0\rho _0}<0$. Then we have

44 $$\begin{aligned} \eta E_0{\tilde{\rho }}_0<\dfrac{\rho _0 k_0}{k_0\rho _0+e^{-k_0\rho _0}-1}. \end{aligned}$$

Consequently, combining with the two conditions (43) and (44), we have the following result:

#### Theorem 6

Assume that the following condition holds

45 $$\begin{aligned} \eta E_0{\tilde{\rho }}_0<\min \{e^{k_0\rho _0},\dfrac{\rho _0 k_0}{k_0\rho _0+e^{-k_0\rho _0}-1}\}. \end{aligned}$$

Then the two equilibria $(F_{--}(\epsilon ), E_{--}(\epsilon ))$ and $(F_{++}(\epsilon ), E_{++}(\epsilon ))$ of model (17) are unstable, while the other two equilibria $(F_{-+}(\epsilon ), E_{-+}(\epsilon ))$ and $(F_{+-}(\epsilon ), E_{+-}(\epsilon ))$ are asymptotically stable.

#### Proof

Using the assumption (45), we can see that the inequality (43) and $\delta >A$ are always true. Note that the sign of *A* is determined by the signs of the following three terms:

$$\begin{aligned} \text {sign}({\tilde{\rho }}_0), ~~\text {sign}(1-k_0\rho _0) ~~\text {and} ~~\text {sign}(\eta E_0{\tilde{\rho }}_0e^{-k_0\rho _0}). \end{aligned}$$

From Fig. 1, we can see that the $\rho (F)$-curve is increasing in the interval (0, *j*) and decreasing for $(j,+\infty )$. When $\epsilon >0$, we have

$$\begin{aligned} \begin{aligned} {\tilde{\rho }}_0>0, ~~ \text {at}~ F_{--}(\epsilon )~ \text {and}~ F_{+-}(\epsilon ),\\ {\tilde{\rho }}_0<0, ~~ \text {at}~ F_{++}(\epsilon )~ \text {and}~ F_{-+}(\epsilon ). \end{aligned} \end{aligned}$$

Moreover, Fig. 1 also shows that

$$\begin{aligned} \begin{aligned} 1-j^{-1}F_0>0, ~~ \text {at}~ F_{--}(\epsilon )~ \text {and}~ F_{+-}(\epsilon ),\\ 1-j^{-1}F_0<0, ~~ \text {at}~ F_{++}(\epsilon )~ \text {and}~ F_{-+}(\epsilon ). \end{aligned} \end{aligned}$$

Since $0<\rho _{-0}<k_0^{-1}<\rho _{+0}$, using a continuity argument, we have

$$\begin{aligned} \begin{aligned} 1-k_0\rho _0>0, ~~ \text {at}~ F_{--}(\epsilon )~ \text {and}~ F_{-+}(\epsilon ),\\ 1-k_0\rho _0<0, ~~ \text {at}~ F_{++}(\epsilon )~ \text {and}~ F_{+-}(\epsilon ). \end{aligned} \end{aligned}$$

Then the sign of *A* is

$$\begin{aligned} \begin{aligned}&\text {sign}(A)<0, ~~ \text {at}~ F_{--}(\epsilon ),\\&\text {sign}(A)>0, ~~ \text {at}~ F_{+-}(\epsilon ),\\&\text {sign}(A)<0, ~~ \text {at}~ F_{++}(\epsilon ),\\&\text {sign}(A)>0, ~~ \text {at}~ F_{-+}(\epsilon ). \end{aligned} \end{aligned}$$

Therefore, $(F_{--}(\epsilon ), E_{--}(\epsilon ))$ and $(F_{++}(\epsilon ), E_{++}(\epsilon ))$ of model (17) are both unstable. The other two equilibria $(F_{-+}(\epsilon ), E_{-+}(\epsilon ))$ and $(F_{+-}(\epsilon ), E_{+-}(\epsilon ))$ are locally asymptotically stable.

$$\square $$

We remark that in the case considered in the above theorem, $\rho (0)=0$ so the trivial equilibrium is always asymptotically stable.

## Simulations and discussions

As shown in previous studies (Brauer 1995; Webb 1985; Metz and Diekmann 2014; Magal and Ruan 2018; Wu 1996; Kosovalić et al. 2017, 2014, 2013), algebraic-delay differential systems arise naturally from the population dynamics, when the individuals are physiologically structured and when the total population in a particular physiological stage is subject to certain dynamics leading to an integral equation with the input flow into the stage acting as a continuous forcing.

Here, we formulated a novel coupled system of a differential equation and an algebraic/integral equation to characterize the tick population dynamics, with a particular focus on the tick attachment/fixation behaviors and host grooming response and the implication of this tick-host interaction on the tick population dynamics at the population level. The model formulation is particularly useful to provide insights of multi-stability in different combinations of tick-host pairs. We did consider two special cases: the case of cooperative feeding and density-dependent grooming when bi-stability occurs, and the case of cooperative feeding and grooming in response to tick biting when quadri-stability becomes a feasible scenario.

We now provide a few numerical examples to show bi-stability and quadri-stability are both possible with parameters suggested from experimental settings or field observations (see Table 1).

**Table 1:** Parameters and their values

Parameters	Baseline values	Baseline values	References
	for bi-stability	for quadri-stability	
$\eta _1$	0.15 (day$^{-1}$)	0.05 (day$^{-1}$)	Hancock et al. (2011)
$\eta _2$	0.3 (day$^{-1}$)	0.1 (day$^{-1}$)	Dunn et al. (2013), Olegario et al. (2011)
$\sigma$	1000 (day$^{-1}$)	300 (day$^{-1}$)	Gaff (2011), Hartemink et al. (2008)
$\tau$	700 (day)	700 (day)	Lindquist and Vapalahti (2008)
*T*	7(day)	7 (day)	Hancock et al. (2011)

*Bi-stability* We start with the case of cooperative feeding and density-dependent grooming, with some parameter values given in the second column of Table 1 and the other parameter values assumed below:

$$\begin{aligned} p=0.002, ~ \delta =0.033, ~ \mu _0=0.4, ~ \mu =0.0014,~ c=100. \end{aligned}$$

**Fig. 2:**
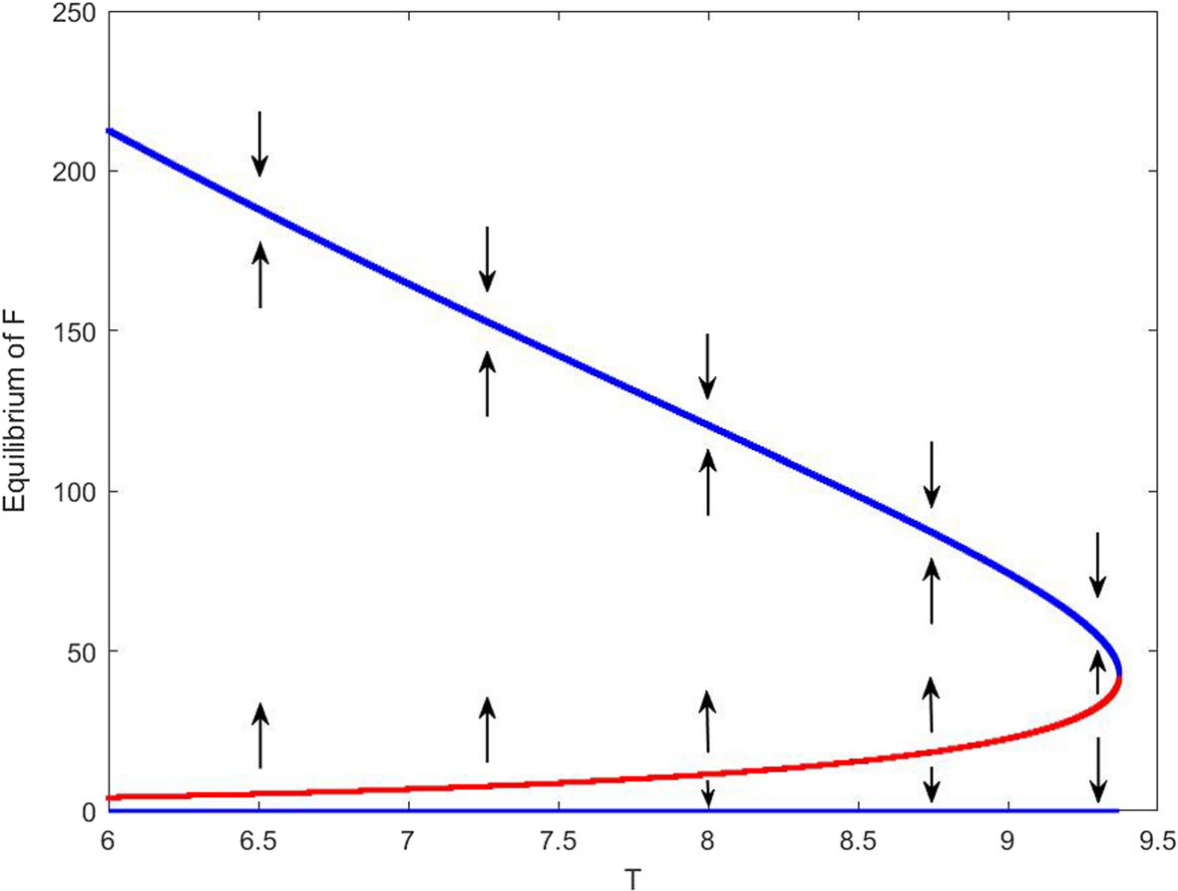
Equilibrium of feeding nymphal ticks with respect to average feeding duration $T\in (6,9.3718)$. For each *T*, model (15) has a stable tick-free equilibrium (bottom blue curve) and two positive equilibria, one stable (top blue curve) and one unstable (middle red curve)

**Fig. 3:**
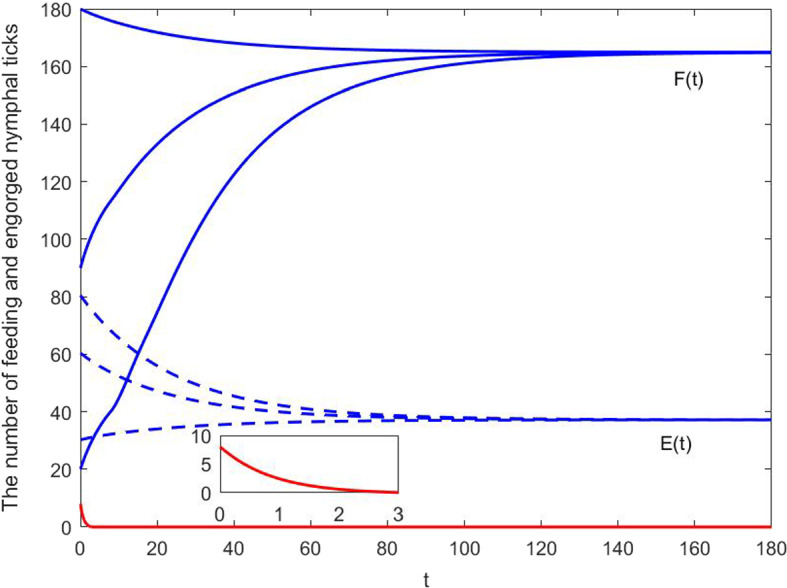
Solutions of engorged and feeding nymphal tick population of model (15) with different initial data: (1) when the initial data are fixed at (80, 180),  (60, 90),   (30, 20) respectively, the numbers of engorged and feeding nymphal ticks approach to $(E_+^*,F_+^*)=(37.1687,164.9135)$; (2) when the initial data is (5, 8), nymphal ticks will go extinct eventually. Model (15) has two locally asymptotically stable equilibria: $(E_+^*,F_+^*)$ and tick-free equilibrium (0, 0)

A simulation is presented in Fig. 2, from which we observe the existence of three equilibria of model (15): one is the tick-free equilibrium $P_0(0,0)$ and the others are positive, the larger positive equilibrium $(E_+^*,F_+^*)=(37.1687,164.9135)$ is stable and the smaller one $(E_-^*,F_-^*)=(5.1798,7.0223)$ is unstable. Illustrated also in Fig. 3 are solutions of feeding nymphal ticks with different initial data that approach two stable equilibria $P_0$ and $(E_+^*,F_+^*)$. Varying the feeding duration $T\in [0,9.3718]$, the two equilibria (in blue) remain to be stable and the middle equilibrium (in red) is unstable, so we have the expected bi-stability and the tick population long-term behaviors depend on the initial condition. If $T^*=9.3718$, model (15) has only one unique positive equilibrium $(E^*,F^*)=(25.9754,135.9141)$, and increasing *T*, the coupled system has the only tick-free equilibrium.

*Quadri-stability* We provide another set of simulations for the case of cooperative feeding and grooming in response to tick biting. Some parameters are listed in the third column of Table 1 and the remained parameters are fixed as follows:

$$\begin{aligned} \delta =0.2,~ k_0=2/7,~ r=0.02,~ j=120. \end{aligned}$$

**Fig. 4:**
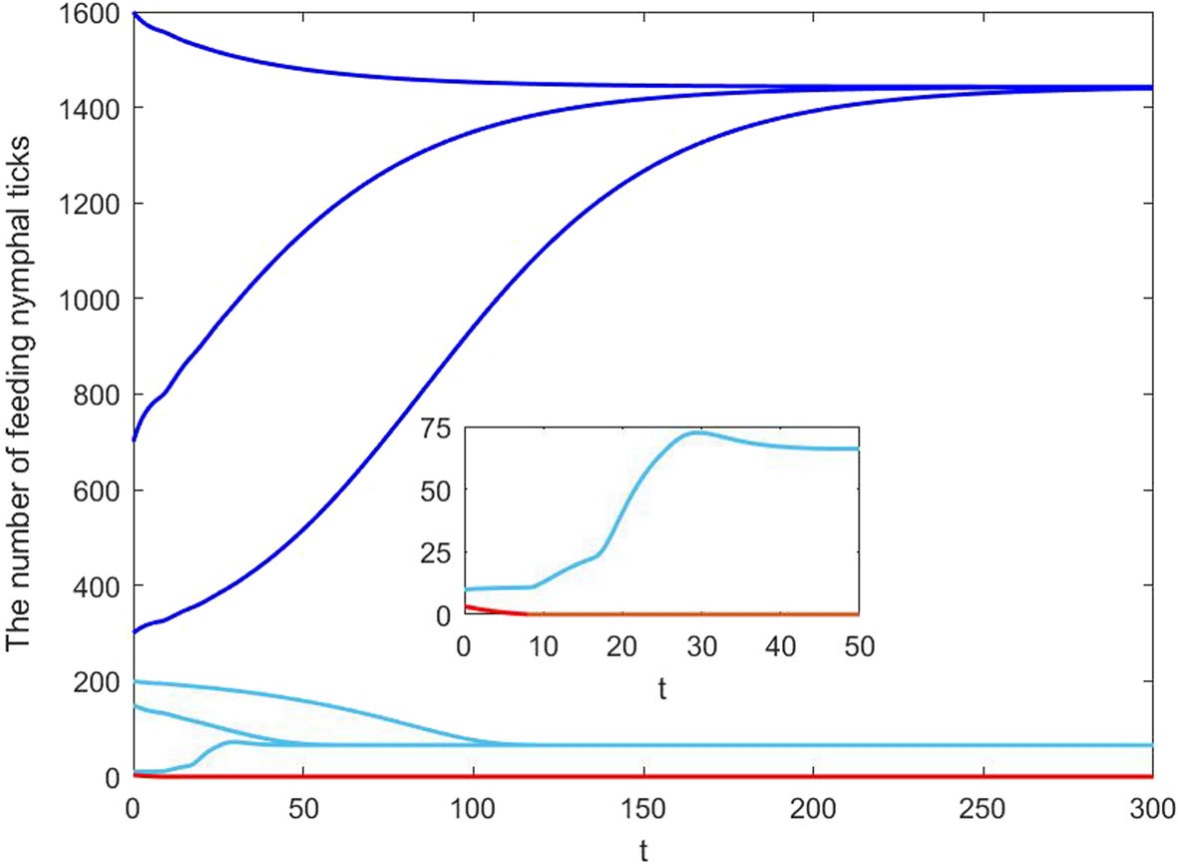
Solutions of feeding nymphal tick population of model (17) with different initial data: (1) when the initial feeding nymphal ticks are set at 1600, 700, 300, the number of feeding nymphal ticks approaches $F_{-+}=1443.8375$; (2) when the initial feeding nymphal ticks are set at 200, 150, 10, the number of feeding nymphal ticks approaches $F_{+-}=65.1540$; (3) when the initial feeding nymphal ticks is 3.3, feeding nymphal ticks will go extinct eventually. Thus, model (17) has three locally asymptotically stable equilibria: $(E_{-+},F_{-+})$, $(E_{+-},F_{+-})$ and tick-free equilibrium (0, 0)

**Fig. 5:**
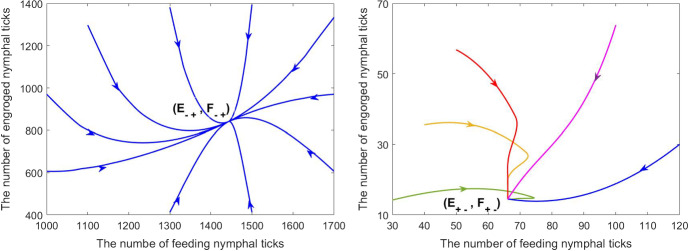
Phase portraits of model (17), with respective curves (*E*(*t*), *F*(*t*)) converging to the two stable positive equilibria: $(E_{-+},F_{-+})$ and $(E_{+-},F_{+-})$

For this set of parameters, we can verify the condition $\delta \eta ^{-1}<(ekT)^{-1}$ ($0.13<0.18$). Then equation (31) has two positive solutions $\rho _{-0}=0.1981$ and $\rho _{+0}=1.0147$. Since $\rho _{+0}<{rj}/{2}=1.2$, model (17) has four positive equilibria $(E_{--},F_{--})=(5.8070,9.9730)$, $(E_{+-},F_{+-})=(14.5072,66.1540)$, $(E_{++},F_{++})=(47.7346,217.6735)$, $(E_{-+},F_{-+})=(840.7008,1443. 8375)$. System (17) has two stable positive equilibria, $(E_{+-},F_{+-})$ and $(E_{-+},F_{-+})$, as shown in Fig. 4. The corresponding phase-portraits are given in Fig. 5.

Due to the incorporation of two time lags (*T* and $\tau$) and the integral term in the algebraic equation for *F*(*t*), local stability analysis of the multiple equilibria coexisted in the coupled system is very difficult and we are only able to conduct the local stability analysis using the perturbation analysis when the feeding duration *T* is relatively small comparing with the long life cycle ($T+\tau$). In the case of cooperative feeding and host grooming in response to tick biting, increasing the feeding duration may lead to nonlinear oscillations around these co-existing equilibria through Hopf bifurcations. In this scenario, obtaining an analytic expression of the critical value of the feeding duration when Hopf bifurcations take place, and describing the global continuation of periodic solutions bifurcated near the corresponding equilibria, remain a challenging task. The global dynamics of such a model has yet to be obtained.

Tick population dynamics and tick-borne disease transmission dynamics have been modeled intensively (see, for example, Gaff and Gross 2007; Lou and Wu 2014; Rosà et al. 2003; Rosà and Pugliese 2007; Wu and Zhang 2020 and references therein). Density-dependent development rates have been empirically estimated using laboratory or field observation, see for example, Ogden et al. (2005). These estimations were used for examining the tick range expansion (Wu et al. 2013). Our contribution here is to use the coupled system to separate the tick attachment/fixation behaviours from the host grooming behaviors in estimating the density-dependent development rates and explore the implication of different combinations of tick attaching and host grooming behaviors. This complements the study of Lou and Wu (2014) on tick seeking assumptions and their implications for disease transmission dynamics.

An important step forward is to expand our coupled system for the tick population dynamics to a coupled system for tick-borne disease transmission dynamics when tick population is further stratified by physiological stages and epidemic status. Separating the tick-attaching and host grooming behaviors for ticks at different stages in describing the tick-borne disease transmission dynamics is also important to understand the co-occurrence of ticks at different stages in the same host, which is critically important to understand the role of co-feeding transmission dynamics (Alekseev and Chunikhin 1990; Hua et al. 2003; Labuda et al. 1993; Mogl et al. 2011; Ogden et al. 1997; Randolph et al. 2002, 1996; Randolph 2011; Wu and Zhang 2021; Zhang et al. 2017).
